# Interparental Relationship Adjustment, Parenting, and Offspring’s Cigarette Smoking at the 10‐Year Follow‐up

**DOI:** 10.1111/famp.12598

**Published:** 2020-09-19

**Authors:** Xiang Zhao, Katharina Prandstetter, Elena Jansen, Kurt Hahlweg, Wolfgang Schulz, Heather M. Foran

**Affiliations:** ^1^ Institute of Psychology University of Klagenfurt Klagenfurt am Wörthersee Austria; ^2^ Department of Clinical Psychology Institute of Psychology Technical University of Braunschweig Braunschweig Germany

**Keywords:** Adolescent smoking, Cigarette smoking, Interparental relationship, Dysfunctional parenting, Smoking progression

## Abstract

Familial influences on children’s cigarette smoking have been established, yet little is known about whether these influences in childhood relate to offspring’s smoking behavior in adolescence. Drawing on prior work showing that children’s emotional and behavioral problems (i.e., internalizing and externalizing behavior problems) are influenced by both interparental and parent–child relationships, we examined whether children’s emotional and behavioral problems would further predict their smoking behavior in adolescence. Two hundred and twenty‐one families were followed from early childhood (*M*_age_ = 4.05 years) to the 10‐year follow‐up. Interparental relationship adjustment and disagreement, dysfunctional parenting, and children’s emotional and behavioral problems were reported by both mothers and fathers. Adolescents’ self‐reported cigarette smoking status was assessed along with other demographic variables. Using structural equation modeling, the hypothesis was only supported based on mothers’ reports, suggesting that early couple relationship adjustment and parenting relate to children’s emotional and behavioral problems, which associate with smoking behavior in adolescence. When the hypothesized model was tested with emotional and behavioral problems separately, only behavioral problems were related to adolescent smoking for both parents. Findings from this study support models of family environment and children’s behavioral problems, providing evidence of the long‐term links with adolescent cigarette smoking behaviors. Further family‐focused research and preventive work, for instance, testing the combination of partner support and parent training, are needed.

## Resumen

Se han establecido las influencias familiares en el consumo de cigarrillos de los niños, sin embargo, se sabe poco acerca de si estas influencias en la niñez se relacionan con la conducta de consumo de cigarrillos de los niños en la adolescencia. Teniendo en cuenta trabajos anteriores que demuestran que los problemas conductuales y emocionales de los niños (p. ej.: los problemas de conductas de exteriorización e interiorización) están influenciados tanto por las relaciones interparentales como por las relaciones entre padres e hijos, analizamos si los problemas emocionales y conductuales de los niños predecirían, además, su conducta de consumo de cigarrillos en la adolescencia. Se siguió a doscientas veintiuna familias desde la primera infancia (edad promedio = 4.05 años) hasta el control a los diez años. Tanto las madres como los padres informaron adaptación y desacuerdo en las relaciones interparentales, crianza disfuncional y problemas conductuales y emocionales de los niños. Se evaluó la situación de consumo de cigarrillos autoinformada por los adolescentes junto con otras variables demográficas. Utilizando modelos de ecuaciones estructurales, se respaldó la hipótesis solo sobre la base de los informes de las madres, lo cual sugiere que la adaptación temprana en la relación de pareja y la crianza se relacionan con los problemas emocionales y conductuales de los niños, los cuales se asocian con la conducta de fumar en la adolescencia. Cuando el modelo hipotetizado se probó con problemas conductuales y emocionales por separado, solo los problemas conductuales se relacionaron con el consumo de cigarrillos de los adolescentes para ambos padres. Los hallazgos de este estudio respaldan los modelos de entorno familiar y los problemas conductuales de los niños, ya que ofrecen indicios de los vínculos a largo plazo con las conductas de consumo de cigarrillos en los adolescentes. Se necesitan más investigaciones centradas en la familia y trabajos preventivos, por ejemplo, la evaluación de la combinación de apoyo para padres y de capacitaciones para padres.

## 摘要

家庭因素对儿童吸烟行为的影响已经得到证实，但对于儿童时期的影响是否与子女青春期的吸烟行为有关，人们知之甚少。以前有一些研究表明，儿童的情绪和行为问题（即内化和外化行为问题）同时受到父母间关系和亲子间关系的影响，鉴于此，本研究调查了儿童的情绪和行为问题会否进一步预测他们在青春期的吸烟行为。从幼儿期（平均年龄为4.05岁）到随后10年的随访中，我们对210个家庭进行了跟踪调查。母亲和父亲都报告了父母间关系的适应与分歧、不良教养以及孩子的情绪和行为问题。青少年自我报告的吸烟状况与其他人口统计变量一起被评估。基于结构方程模型的分析发现，这一假设仅基于母亲报告的数据，表明早期夫妻关系的适应和养育与孩子的情感和行为问题有关，而这些问题与青春期的吸烟行为有关。当假设的模型分别用情感问题和行为问题进行测试时，只有行为问题与父母双方的青少年吸烟有关。本研究结果支持家庭环境与儿童行为问题的模型，为青少年吸烟行为的长期关联提供了证据。未来研究需要进一步开展以家庭为重点的研究和预防工作，例如研究伴侣支持和父母培训两者的结合。

Europe has the largest age‐standardized cigarette smoking prevalence compared to other world regions (World Health Organization, 2018), and Europe’s smoking prevention is in its early stages. Longitudinal studies show that people who smoke in their adolescence are more likely to become an established smoker in adulthood (Merline, O’Malley, Schulenberg, Bachman, & Johnston, 2004) and to consume more cigarettes (Morrell, Song, & Halpern‐Felsher, 2011). Although adolescence appears to be a suitable period for antismoking interventions, most well‐designed programs targeting this cohort were undertaken in North America (Thomas, McLellan, & Perera, 2013). Apart from the inadequate attempts to prevent smoking, existing smoking interventions have low ecological validity. Individual‐centered behavioral models have been widely applied in antismoking programs, but such models are limited in addressing broader, societal or familial influences on smoking behaviors (Thomas et al., 2013; Zhao, Young, & White, 2019).

Familial influences have been identified as an important etiology for adolescent smoking (Tyas & Pederson, 1998). Nevertheless, existing research mostly used cross‐sectional designs to examine the associations between adolescent smoking and family variables such as parental smoking status, parental supervision, cigarette availability, and parenting styles (Chassin et al., 2005; Koetting O’Byrne, Haddock, & Poston, 2002; Tyas & Pederson, 1998). This static methodological perspective fails to capture the dynamics within the family. From a family ecological viewpoint, adolescents’ substance use may be an attempt to cope with familial systematic changes such as interparental disagreement or divorce (Klostermann, 2014). Therefore, relationships of family members could be the target of antismoking interventions. However, research on this plausible mechanism is currently limited.

Another knowledge gap in adolescent smoking is the transition from childhood to adolescence. Although it is known that most people across countries become smokers between 15 and 24 years old (World Health Organization, 2018), the extent to which one’s earlier familial influences in childhood impact on one’s later tobacco use in adolescence is under‐researched. As most longitudinal studies on smoking focused on the adolescence‐to‐adulthood transition period (Bauman et al., 2002; Biglan, Duncan, Ary, & Smolkowski, 1995; Chassin, Presson, Rose, & Sherman, 1996; Costello, Dierker, Jones, & Rose, 2008; Fosco & Feinberg, 2018; Russek & Schwartz, 1997), the role of parenting in childhood has been largely overlooked. Taken together, more research on the relationship between family processes and adolescent smoking is needed. While previous studies investigated familial risk factors of adolescent smoking mostly from an individualistic perspective, research on associations between early childhood family dynamics and adolescent smoking will fill a gap in the literature.

## The Current Study: From Interparental Relationship Adjustment and Parenting to Offspring’s Future Smoking

It is widely known that interparental relationship adjustment, also referred to as marital conflict, is associated with parenting quality and children’s emotional and behavioral problems which, in turn, influence a wide range of functioning among children (Cummings & Davies, 1994, 2002). A plethora of studies have demonstrated how interparental and parent–child systems impact on child emotional and behavioral problems (Cummings & Davies, 2002; Cummings, Goeke‐morey, & Papp, 2003). For instance, O'Leary and Vidair (2005) found that child‐rearing disagreement (i.e., parent arguments about responses to child misbehavior) mediated the relationship between marital conflict and child behavior problems in children aged 3–7 years, while overactive discipline was associated with child behavior in most models. Similar to a wide range of other studies (Cheung, Cummings, Zhang, & Davies, 2016; Gao & Cummings, 2019; Krishnakumar & Buehler, 2000; Osborne & Fincham, 1996; Sandler et al., 2012), distinctions between mother–child and father–child relationships were found. For instance, a longitudinal study found that interparental relationship adjustment and child‐rearing difficulty (e.g., parental psychological control) were only significantly related in the paternal pathway (Davies, Sturge‐Apple, Woitach, & Cummings, 2009). The assumption that marital conflict affects all parent–child dyads in a similar fashion has been challenged since the 1990s with data from a review (Phares & Compas, 1993) showing that variance in child adjustment that was not captured by mother‐related variables was explained by father‐related variables such as the father–child relationship. While mothers and fathers play their roles in interparental relationship adjustment, both may be utilizing different conflict strategies (Goeke‐Morey, Cummings, Harold, & Shelton, 2003). Belsky, Gilstrap, and Rovine (1984) and later researchers (Cummings, Goeke‐Morey, & Raymond, 2004) postulated the fathering vulnerability hypothesis which indicates that the father–child relationship or fathers’ parenting is susceptible to and therefore more adversely affected by interparental relationship adjustment and other family stressors. For instance, Howes and Markman (1989) suggested that fathers may be more likely to withdraw from their wives and thus their children if they experience interparental conflict or an unhappy marriage. Withdrawal within the father–child relationship may consequently lead children to feel sad, rejected or unloved which could be reflected in emotional problems.

Although these parent–offspring interactions in the family show a stable association on children’s conduct, only recently have researchers specifically focused on how family functioning potentially influences adolescent smoking. Family dynamics characterized by openness and clarity of expression may have a preventive function for adolescent smoking (Koetting O’Byrne et al., 2002), while interparental threat perceived by adolescents predicts their escalation of tobacco use (Fosco & Feinberg, 2018). Given the cross‐sectional or short‐term longitudinal designs, previous research failed to examine how family functioning relates to children’s smoking in a long term. In addition, the existing research often relied only on adolescent self‐report rather than multiple informants. Finally, given the previous focus of studies on influences of interparental and parent–child relationships on child adjustment, examination of child adjustment (i.e., emotional and behavioral problems) as a mediator between these family processes and adolescent smoking seems warranted.

To address the research gaps in the smoking literature, we draw on prior work from biopsychosocial family models including emotional security theory (Davies & Cummings, 1994), spillover theory (Erel & Burman, 1995), and risky family model (Repetti, Taylor, & Seeman, 2002) as these theories consider family dysfunction as a key risk factor for the development of mental and physical health problems in children. Following these theoretical frameworks, children’s emotional and behavioral problems are largely influenced by the quality of interparental as well as parent–child relationships (Cummings, Schermerhorn, Davies, Goeke‐Morey, & Cummings, 2006; Krishnakumar & Buehler, 2000). Several studies have highlighted the importance of dysfunctional parenting practices as a mediator of the relationship between interparental disagreement and child maladjustment, assuming that destructive parental conflicts shape negative parenting behavior, which, in turn, may promote children’s behavioral problems (Buehler & Gerard, 2002; Cummings & Davies, 2010; Gerard, Krishnakumar, & Buehler, 2006; Kaczynski, Lindahl, Malik, & Laurenceau, 2006; Schoppe‐Sullivan, Schermerhorn, & Cummings, 2007).

**Figure 1:**
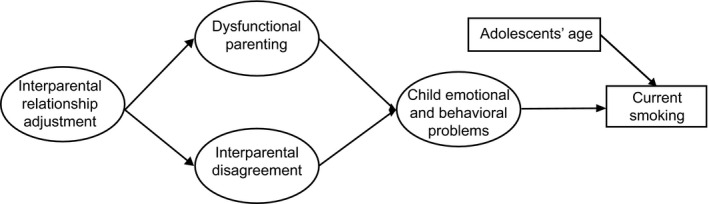
A hypothetical model predicting adolescents’ current smoking.

A dysfunctional family environment, featuring malfunctioning in interparental and parent–child relationship, may trigger children’s substance abuse including cigarette smoking (Repetti et al., 2002). Our study, therefore, builds upon biopsychosocial family models by examining the association between interparental relationship adjustment and adolescent’s current smoking status in a longitudinal research design. As a first step, we hypothesized that dysfunctional parenting practices and interparental disagreement in child‐rearing serve as mediating variables for the association between interparental relationship adjustment and child emotional and behavioral problems. Secondly, we hypothesized that emotional and behavioral problems during childhood relate to smoking in adolescence (Figure 1). Using a 10‐year follow‐up design, this study aimed to extend existing knowledge about family functioning and children’s smoking behavior by proposing a model that tests for the interplay of these variables in a multiple informants and longitudinal framework. Since mothers and fathers potentially have distinctive parent–child relationship patterns, the hypothesized model was examined separately.

## Method

### Procedure

Families in this study were invited to participate in a universal prevention program targeting parenting skills (i.e., Triple P—Positive Parenting program; for details, see Heinrichs, Bertram, Kuschel, & Hahlweg, 2005; Sanders, 2012). The recruitment was conducted in city‐funded preschools in Braunschweig, a moderate size city in Germany. From initially 23 preschools that expressed interest for further involvement, 17 were randomly selected to participate and the project was presented to the families. To adjust for socioeconomic status, the selection of these preschools was based on the kindergarten social structure index (Bäse, 1995). The eligibility criteria for parents included (1) fluency in German; and (2) being the caregiver of a preschool child aged 2.5–6 years. The majority of families participating in the program were German with approximately 11% of the overall sample coming from another country (primarily Turkish background). Only data of parents who were cohabitating or married were included in the current study so that interparental relationship adjustment and conflict at baseline could be examined.

Based on a randomized assignment, nearly half (47.8%) of the families in our sample received a brief parenting intervention at baseline as part of the experimental condition (for details, see Heinrichs et al., 2005). Parents allocated to the control group were not provided with any training and changes were naturally observed over the time of data collection. Participants were assessed six times during the 10‐year study (baseline, four additional times every 12 months after the pre‐assessment, and 10 years after the baseline). Data from baseline (Wave 1), at each yearly interval (Waves 2–5), and at the 10‐year follow‐up (Wave 6) were included in the current analyses. Most participants (91.3%) completed surveys across the ten years (221 of 242 families), with more complete maternal data (*n* = 212) than paternal data (*n* = 187). Fifty Euros were given to participate in the initial assessment; 20 Euros for all subsequent assessments, and 40 Euros for the 10‐year follow‐up participation. Project staff conducted home visits where parents were asked to complete project‐related self‐report measures independently. This study was approved by the ethics committee (German Society for Psychology, WS 12_2010), and informed consent of participants was obtained.

### Participants

**Figure 2:**
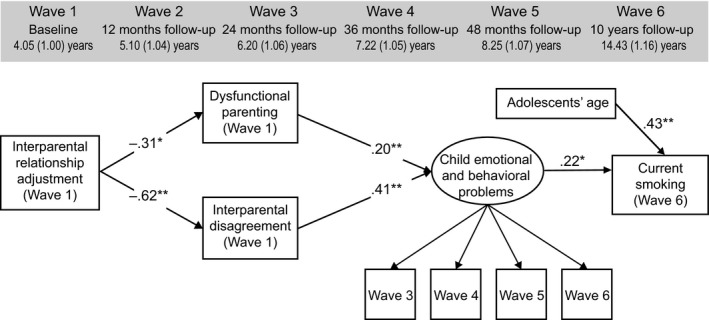
A model predicting adolescents’ current smoking at 10‐year follow‐up based on their mothers’ reports (*n* = 212). Child emotional and behavioral problems were assessed using the total score of the CBCL. An alternative model assuming a relationship between interparental disagreement and dysfunctional parenting was also tested and is reported in the Appendix. For models based on respective scores of CBCL emotional and behavioral problems, see the Appendix. Waves 1 and 2 data of child emotional and behavioral problems were excluded as a different version of the CBCL was used. Standardized coefficients are reported. Model fit: RMSEA = .01; CFI/TLI = .99/.99; WRMR = .03. *p < .05. **p < .01.

**Figure 3:**
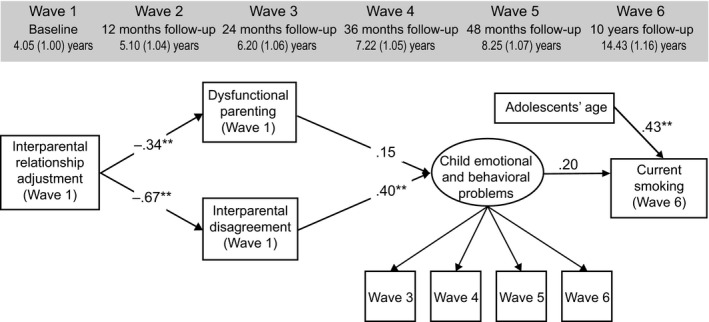
A model predicting adolescents’ current smoking at 10‐year follow‐up based on their fathers’ reports (*n* = 187). Child emotional and behavioral problems were assessed using the total score of the CBCL. An alternative model assuming a relationship between interparental disagreement and dysfunctional parenting was also tested and is reported in the Appendix. For models based on respective scores of CBCL emotional and behavioral problems, see the Appendix. Waves 1 and 2 data of child emotional and behavioral problems were excluded as a different version of the CBCL was used. Standardized coefficients are reported. Model fit: RMSEA = .05; CFI/TLI = .97/.96; WRMR = .05. **p < .01.

A sample of 221 families was obtained with average ages of 38.53 (*SD* = 5.51) years for fathers and 35.30 (*SD* = 4.74) years for mothers. All participants were in opposite‐sex relationships. Children’s mean age was 4.05 (*SD* = 1.00) years at Wave 1 (ranging from 2.5 to 6 years; for specific age information at each subsequent wave, see Figures 2 and 3) and nearly half were girls (46.7%). Most families (54%) received a middle income (i.e., 1,500–3,000 Euros per month post‐tax); 34% of the sample reported an income over 3,000 Euro; 11% reported an income of 1,500 Euro or less; and the rest chose not to report their income. Full‐time employment was reported by 79% of fathers and 10% of mothers; 2% of fathers and 46% of mothers reported part‐time employment; and 8% of fathers and 43% of mothers were unemployed. In terms of education level, more than half of the surveyed (fathers 57%, mothers 58%) were highly educated (i.e., had attended at least college according to the German educational system). Most couples (73.7%) retained the same marital status over the 10‐year period (i.e., from Wave 1 to Wave 6). The parents who participated at the 10‐year follow‐up were living with the child. There was no difference between couples who remained in the study and those who dropped out in terms of child gender, χ^2^_(1)_ = 2.87, *p* = .140, child smoking status at 10‐year follow‐up, χ^2^_(1)_ = .25, *p* = .644, or child emotional and behavioral problems reported by mothers, *F*(1, 186) = .07, *p* = .793 and fathers, *F*(1,245) = .13, *p* = .724 at baseline.

### Measures

#### Adolescent current smoking (Wave 6)

One item was used to assess adolescents’ cigarette smoking status at the last wave (i.e., “Do you currently smoke cigarettes?”), using a 4‐item response scale (“No,” “Daily,” “Several times a week,” and “Less common”). Smoking was only assessed at Wave 6 because the participants were too young to be expected to smoke in previous waves. Given the non‐normality of this score (kurtosis = 16.07, skewness = 4.07), it was recoded to compare adolescents who ever (8.4%) or never (91.6%) smoked cigarettes, as suggested by Tabachnick and Fidell (2013).

#### Child emotional and behavioral problems (Waves 3 to 6)

The German version of the Child Behavior Checklist (Achenbach & Rescorla, 2000; Döpfner et al., 1998) was used to assess emotional and behavioral problems (also termed as internalizing and externalizing symptoms) in children. Data from Waves 3–6 were selected because the 4–18‐year version of the CBCL was used consistently for these waves, whereas previous waves used the 1.5–5‐year version. Parents were presented 118 items and asked to rate the frequency of certain behaviors of their children on a 3‐point Likert scale (e.g., “Destroys things belonging to his/her family or other children”; 0 [*not true*]; 1 [*somewhat or sometimes true*]; 2 [*very true or often true*]). Besides the total score, with higher scores indicating higher emotional and behavioral problems in children, subscales for emotional and behavioral problems were also examined. The combined version of the CBCL showed excellent internal consistencies at Wave 3 (α_mother_ = .95, α_father_ = .92); internal consistencies for behavioral (α_mother_ = .91; α_father_ = .90) and emotional problems (α_mother_ = .84, α_father_ = .74) were also acceptable; Wave 6 values for the combined version (α_mother_ = .95, α_father_ = .95)—as well as behavioral (α_mother_ = .92; α_father_ = .92) and emotional (α_mother_ = .88, α_father_ = .87) problems—were satisfactory.

#### Dysfunctional parenting (Wave 1)

The German version (Naumann et al., 2010) of the Parenting Scale (PS; Arnold, O'Leary, Wolff, & Acker, 1993) was used to assess dysfunctional parenting practices (i.e., overreactivity, laxness, and verbosity). The self‐report questionnaire included 35 items with a 7‐point Likert scale (from 1 [*most effective*] to 7 [*most ineffective*]). Parents were asked to choose from two contrasting situations (e.g., *I am the kind of parent that*… 1 [*sets limits on what my child is allowed to do*]; 7 [*lets my child do whatever he/she wants*]). Items were summed up to a total score, with higher scores reflecting more dysfunctional parenting. The scale showed satisfactory internal consistency indices (α_mother_ = .87, α_father_ = .86).

#### Interparental disagreement (Wave 1)

In order to assess interparental disagreement in child‐rearing, the German version (Kröger, Hahlweg, Heinrichs, Döpfner, & Plück, 2009) of the Parent Problem Checklist (PPC; Dadds & Powell, 1991) was used. The 16‐item questionnaire examined the conflict over child misbehavior rules as well as parents’ ability to work as a team in parenting. Parents were asked to indicate on a 4‐point Likert scale (1 [*not true*]; 2 [*rather not true*]; 3 [*rather true*]; 4 [*true*]) whether the following problems had occurred (e.g., disagreement about rules in the house about sleeping time or where to play) in the last four weeks. Items were summed and averaged as indicators of interparental disagreement. The PPC showed satisfactory reliability at Wave 1 (α_mother_ = .84, α_father_ = .85).

#### Interparental relationship adjustment (Wave 1)

The German version (Köppe, 2001) of the Abbreviated Dyadic Adjustment Scale (ADAS; Sharpley & Rogers, 1984) was utilized to measure the quality of couple’s romantic relationship. Parents rated how much they agreed on several values (e.g., philosophy of life) and interparental interactions (e.g., “Do you and your partner make future plans together?”) on a 6‐point Likert scale. Finally, parents were asked to indicate how satisfied they were with their current relationship. All items were summed up to a total score, with higher scores indicating better relationship adjustment. Internal reliability for mothers (α = .80) and fathers (α = .81) was satisfactory.

#### Covariates

Adolescent sex and age, parental marital status changes over 10 years, and intervention condition (prevention condition vs. control condition) were also assessed.

### Data analysis

Descriptive and correlational analyses were conducted first. Data from mothers and fathers were analyzed separately, given the differences identified previously (Gao & Cummings, 2019). Structural equation modeling was subsequently performed to examine relationships among study variables (see Figures 2 and 3). The non‐normal distribution of the outcome variable necessitated the use of the weighted least square mean and variance adjusted (WLSMV) estimator, which is suitable for non‐normally distributed variables. WLSMV is also able to treat missing data using Mplus 8.2 (Asparouhov & Muthén, 2010). Four criteria were used to evaluate good model fit: Comparative Fit Index (CFI) ≥ .95, Tucker–Lewis Index (TLI) ≥ .95, root mean square error of approximation (RMSEA) ≤ .05, and the weighted root mean square residual (WRMR) ≤ .90 (Hu & Bentler, 1999; Yu, 2002).

Since older adolescents are more likely to smoke compared to younger adolescents (Fuemmeler et al., 2013), age was considered as an exogenous variable for adolescent smoking. Moreover, it is necessary to consider the existence of effects of adolescents’ sex, parental marriage status changes over time, and intervention condition; thus, we controlled for these covariates in the proposed model. Specifically, the associations between adolescent smoking and these covariates were initially examined; only covariates showing a significant association with smoking were kept in the final model. Similar tests were also performed to examine the associations between CBCL scores (i.e., constructed as a latent variable indicated by Waves 3 to 6 data) and the above‐mentioned covariates; no covariate showed a significant relation with the CBCL scores.

The CBCL was administered at earlier time points (i.e., Wave 1 and Wave 2) using a different version (i.e., 1.5–5‐year version). To avoid discrepancies between the measurement versions, only data based on the 4‐18‐year version were used in this study. Moreover, from a developmental perspective, ages across Wave 3 to Wave 6 belong to school‐aged childhood (Sawyer, Azzopardi, Wickremarathne, & Patton, 2018).

## Results

### Descriptive Analysis

Descriptive statistics and correlations are presented in Table 1. Among the covariates, only adolescents’ age was positively correlated with their current smoking status (ρ = .23, *p* < .01) and was, therefore, kept in the final model. Children’s emotional and behavioral problems (i.e., CBCL total scores), dysfunctional parenting, interparental disagreement, and interparental relationship adjustment were all significantly intercorrelated. Parents’ reports on these variables were compared, only finding that mothers reported more interparental disagreement than fathers (*M*_mother_ = 3.71, *M*_father_ = 3.18; *t*_(186)_ = 2.10, *p* = .04).

**Table 1:** Correlations, Means, and Standard Deviations of Study Variables Based on Mother and Father Report

Variable	1	2	3	4	5	6	7	8
1. Current smoker—adolescent (Wave 6)	–	.23**	.00	.09	.02	.14	.05	.02
2. Age (Wave 6)	.23**	–	−.06	.08	.02	.08	.03	−.09
3. Sex (male)	−.00	−.06	–	−.03	−.02	.01	−.08	.07
4. Intervention condition	.09	.076	−.03	–	.02	.04	.01	.00
5. Child emotional and behavioral problems (Wave 3)	.08	.04	.09	.04	–	.20**	.37**	−.29**
6. Dysfunctional parenting (Wave 1)	.15*	.023	−.05	.05	.28**	–	.43**	−.23**
7. Interparental disagreement (Wave 1)	.06	.049	−.03	.13	.36**	.30**	–	−.55**
8. Interparental relationship adjustment (Wave 1)	−.04	−.11	−.00	.03	−.29**	−.24**	−.56**	–
Mean (m)	8.4%	14.43	53.3%	66.3%	22.34	3.23	3.71	22.75
*SD* (m)		1.16			18.30	.56	3.39	5.08
Mean (f)					17.63	3.22	3.18	23.21
*SD* (f)					13.86	.46	2.92	5.00

Note

Numbers under the diagonal reflect mothers’ report (m), whereas numbers above the diagonal are based on fathers’ data (f). Correlations related to categorical variables 1, 2, 3, and 4 were examined using Spearman’s ρ, other continuous variables using Pearson’s *r*. Given the high test–retest reliability, only Wave 3 scores on child emotional and behavioral problems were used in this table for brevity.

* *p* < .05.

** *p* < .01. Child emotional and behavioral problems = CBCL total scores.

### Associations with Smoking of Offspring

As shown in Figures 2 and 3, both final models yield acceptable to good fit as evidenced by fit statistics (maternal model: RMSEA = .01; CFI/TLI = .99/.99; WRMR = .03: paternal model: RMSEA = .05; CFI/TLI = .97/.96; WRMR = .05). In both models, lower interparental relationship adjustment was related to more dysfunctional parenting and interparental disagreement, which, in turn, was associated with more child emotional and behavioral problems. The link between dysfunctional parenting and child emotional and behavioral problems also showed a positive relationship at a near‐significant level (*B* = .15, *p* = .076). Older adolescents were more likely to experiment with smoking. Interestingly, although child emotional and behavioral problems perceived by both mothers and fathers showed a positive relationship with their offspring’s smoking status at the 10‐year follow‐up, this coefficient was only significant in the maternal model (*B* = .22, *p* = .034). This relationship showed no significant difference between maternal and paternal models in further multigroup comparison, χ^2^_(1)_ = .04, *p* = .838.

Models based on child emotional and behavioral problems rather than the total score were also tested given the common use of these subscales in family psychological research (Mulvaney & Mebert, 2007; Teubert & Pinquart, 2010; Warmuth, Cummings, & Davies, 2020) as well as their established associations with smoking behavior (Colder et al., 2013; Jester et al., 2019). Consistent with the models using the total score, both emotional and behavioral models showed a good fit (see Appendix). While the models using CBCL behavioral problem scores showed a significant prediction of adolescent smoking for both mothers and fathers, such prediction was nonsignificant in the CBCL emotional problem models (see Appendix for more model results as well as the alternative models, one of which additionally includes the relationship between disagreement and parenting).

## Discussion

Early familial vulnerability for children’s later substance use has been identified as a research gap (Chassin et al., 2016). Using a longitudinal design, this study tested how one’s emotional and behavioral problems associate with one’s cigarette smoking in adolescence. As a result, the combined score of child emotional and behavioral problems reported by mothers showed a positive link with adolescents’ smoking experience 10 years later. By contrast, the models using CBCL behavioral problem scores showed a significant association with adolescent smoking for both mothers and fathers. Our findings have extended the existing knowledge about adolescent smoking and interparental relationship (Fosco & Feinberg, 2018), underscoring the long‐term association between early interparental relationship adjustment, parenting, and child behavioral problems. The indirect link between early childhood family process factors and cigarette smoking behavior of the child 10 years later is also discerned.

Consistent with a previous longitudinal study tracing participants from 12 to 21 years (Guo, Hill, Hawkins, Catalano, & Abbott, 2002), findings from this study highlight that family/interparental disharmony serves as a detrimental factor for the offspring’s substance use. Results are consistent with the biopsychosocial family models in which early family environment impacts children’s mental and physical health (Cummings & Davies, 2002). This study adds to the literature by examining these relationships from early childhood into adolescence, especially considering the influence of family process in adolescent smoking.

With a multiple informants’ perspective, our study confirms this developmental pattern based on maternal report but not paternal report. Consistent with the finding that mothers observe more offspring problems than fathers (Baker & Heller, 1996), our findings only identified the significant link between child emotional and behavioral problems and adolescent smoking in the maternal model. While the above link including the total score lacked a significant difference in multigroup comparison between mothers and fathers, separate analyses by problem type (see Appendix) indicated that behavioral problems rather than emotional problems were associated with adolescent smoking behavior based on reports by both mothers and fathers. Although the study had a relatively high level of father participants followed over a 10‐year period, the father sample size was smaller than the sample size for mothers; this fact may explain why the significant link in the main analysis was only found in the maternal model.^1^ Future studies with more equal sample sizes can help further unpack these findings.

Previous family‐based programs targeting adolescent smoking have shown successful outcomes (Bauman et al., 2002). Our findings suggest that interventions in early childhood acknowledging the couple relationship and parenting may be relevant for curbing the offspring’s future smoking attempts. Dadds, Schwartz, and Sanders (1987) have shown benefits of a program combining parent training and partner support training on child outcomes such as behavior problems and deviance. As noted by Chassin et al. (2016), family‐based interventions could generate preventive benefits on offspring’s substance use including tobacco and alcohol use initiation and clinical addictive problems. Nonetheless, robust family‐based programs specifically targeting smoking are still limited (Rohrbaugh et al., 2001). However, as our results indicated, family‐based interventions focusing on couple relationships and parenting abilities could aim at general health promotion rather than substance‐specific prevention, because child behavioral problems appeared to be the direct "precursor" of adolescent smoking in the model. By enhancing students’ social skills, a well‐established antidrug program initiated in the U.S. (Botvin & Griffin, 2015) reported its preventive effect for multiple types of substance use (e.g., alcohol, tobacco, marijuana) among children and adolescents. Thus, family‐based interventions could achieve a similar preventive outcome on substance use by eliminating child’s emotional and behavioral problems via better parenting skills and interparental relationship adjustment.

The present study includes several limitations. Adolescent smoking experience over a 10‐year period may be mediated by other factors such as media (Davey & Zhao, 2012), peer influences which potentially co‐occur with family conflicts (e.g., children may spend more time on screen media or with friends if their family climate is disharmonious), parental smoking status across time, and concurrent individual affective disorders such as depression (Chen, Rittner, Maguin, & Dziadaszek, 2016). Marital status changes of couples were not assessed at each time point, and other dynamics within the family may have also played a role that is beyond the scope of this study. The measure of tobacco used in this study may not capture the explorative nature of smoking action among adolescents (Davey & Zhao, 2020). Although the relatively low smoking prevalence is similar to the national status (Orth & Merkel, 2018), smoking incidences may be underestimated due to the self‐report nature of the measure used (Ma et al., 2014). Moreover, given the emerging forms of smoking (e.g., e‐cigarettes), a broader measure of tobacco use may remedy the low smoking prevalence observed in this study. Relatively small sample sizes with multiple time points also limited the use of dyadic modeling or testing of more complex longitudinal models. Future studies could explore the longitudinal patterns of familial variables using latent growth curve models or other methods and their influences on adolescent smoking. While our study incorporated both parents’ perspective on child emotional and behavioral problems over 10 years, it would also be important to assess this information from a child‐based perspective, for instance, by applying the Youth Self‐Report Form (Achenbach, 1991). However, by using a multi‐informant, 10‐year longitudinal design across childhood to adolescence, our study has filled a void in the literature highlighting the role of familial factors in offspring’s later tobacco use. Findings from this study call for more family‐focused tobacco use research.

## Footnotes

^1^ We tested the model with a combined approach based on worse scores (i.e., using the worse score reported by a couple; see Appendix). Given the large influence from the mothers’ report due to the larger sample size of mothers, this approach was deemed inappropriate.

## Appendix

**Table A1:** Summaries of Models Using CBCL Emotional and Behavioral Problems Scores Separately

Model path	Mother—Emotional problems (Model fit: RMSEA = .04; CFI/TLI = .98/.97; WRMR = .05)	Mother—Behavioral problems (Model fit: RMSEA = .03; CFI/TLI = .98/.98; WRMR = .05)	Father—Emotional problems (Model fit: RMSEA = .03; CFI/TLI = .98/.97; WRMR = .04)	Father—Behavioral problems (Model fit: RMSEA = .05; CFI/TLI = .96/.95; WRMR = .06)
CBCL ON				
Dysfunctional parenting	*B* = .14, *p* = .004	*B* = .22, *p* < .001	*B* = .12, *p* = .087	*B* = .19, *p* = .018
Interparental disagreement	*B* = .39, *p* < .001	*B* = .34, *p* < .001	*B* = .43, *p* < .001	*B* = .36, *p* < .001
Smoking ON				
CBCL	*B* = .06, *p* = .637	*B* = .32, *p* = .002	*B* = .08, *p* = .604	*B* = .29, *p* = .015
Smoking ON				
Adolescents’ age	*B* = .43, *p* < .001	*B* = .43, *p* < .001	*B* = .43, *p* < .001	*B* = .43, *p* < .001
Dysfunctional parenting ON				
Interparental relationship satisfaction	*B* = −.31, *p* < .001	*B* = −.31, *p* < .001	*B* = −.33, *p* < .001	*B* = −.33, *p* < .001
Interparental disagreement ON				
Interparental relationship satisfaction	*B* = −.65, *p* < .001	*B* = −.62, *p* < .001	*B* = −.69, *p* < .001	*B* = −.64, *p* < .001

Note

CBCL = child emotional and behavioral problems.

Standardized path coefficients (*B*) are reported. Different from the main study, analyses reported here used the subscales (i.e., emotional and behavioral problems) instead of a total score.

**Table A2:** Summaries of the Worse Score Model Outcomes

Model path	Coefficient
CBCL ON	
Dysfunctional parenting	*B* = .18, *p* = .001
Interparental disagreement	*B* = .37, *p* < .001
Smoking ON	
CBCL	*B* = .23, *p* = .031
Smoking ON	
Adolescents’ age	*B* = .43, *p* < .001
Dysfunctional parenting ON	
Interparental relationship satisfaction	*B* = −.44, *p* < .001
Interparental disagreement ON	
Interparental relationship satisfaction	*B* = −.69, *p* < .001

Note

CBCL = child emotional and behavioral problems.

The total score of emotional and behavioral problems was used for this worse score model. Model fit: RMSEA = .03; CFI/TLI = .99/.99; WRMR = .04. Standardized path coefficients (*B*) are reported. Different from the main study, analyses reported here used a worse score approach based on the CBCL total score. A worse score means the smaller/larger (the direction depends on the meaning of the scale) number (either from the mother or the father) reported in a couple.

**Table A3:** Summaries of Alternative Models Hypothesizing the Relation between Interparental Disagreement and Dysfunctional Parenting

Model path	Mother model (Model fit: RMSEA = .03; CFI/TLI = .99/.99; WRMR = .04)	Father model (Model fit: RMSEA = .03; CFI/TLI = .99/.98; WRMR = .04)
CBCL ON		
Dysfunctional parenting	*B* = .20, *p* = .003	*B* = .02, *p* = .831
Interparental disagreement	*B* = .33, *p* < .001	*B* = .46, *p* < .001
Smoking ON		
CBCL	*B* = .22, *p* = .045	*B* = .20, *p* = .122
Smoking ON		
Adolescents’ age	*B* = .39, *p* = .001	*B* = .43, *p* < .001
Dysfunctional parenting ON		
Interparental relationship satisfaction	*B* = −.10, *p* = .109	*B* = .11, *p* = .411
Interparental disagreement	*B* = .20, *p* = .021	*B* = .53, *p* < .001
Interparental disagreement ON		
Interparental relationship satisfaction	*B* = −.11, *p* < .001	*B* = −.64, *p* < .001

Note

CBCL = child emotional and behavioral problems.

The total score of emotional and behavioral problems was used for the above models. Standardized path coefficients (*B*) are reported. Different from the main study, analyses reported here further hypothesized a relationship between dysfunctional parenting and interparental disagreement.
